# Physical Activity Monitoring Preferences in Adults With Bipolar Disorder

**DOI:** 10.3389/fpsyt.2021.657043

**Published:** 2021-07-22

**Authors:** Carol A. Janney, Abigail R. Ducheine, Robert Reichmann, Matthew A. Stack, Andrea Fagiolini

**Affiliations:** ^1^Director of Research, Pine Rest Christian Mental Health Services/Assistant Professor in the Division of Psychiatry and Behavioral Medicine, and Department of Epidemiology and Biostatistics at the Michigan State University College of Human Medicine, Grand Rapids, MI, United States; ^2^University of Pittsburgh Medical Center, Pittsburgh, PA, United States; ^3^Core Faculty at Michigan State University/MidMichigan Medical Center - Gratiot Family Medicine Residency, Alma, MI, United States; ^4^MidMichigan Health, Shepherd, MI, United States; ^5^Faculty for Behavioral Health at Michigan State University/MidMichigan Medical Center - Gratiot Family Medicine Residency, Alma, MI, United States; ^6^Professor of Psychiatry, Department of Molecular Medicine, University of Siena School of Medicine, Siena, Italy

**Keywords:** actigraphy, pedometer, accelerometry, physical activity, bipolar disorder, patient-centered, smi

## Abstract

This report investigated physical activity (PA) monitoring preferences and problems among adults with bipolar disorder (BD).

**Methods:** PARC2 study was conducted at the Western Psychiatric Institute and Clinic at the University of Pittsburgh. This secondary data analysis assessed three PA monitors; Body Media SW Pro Armband, Actigraph AM-7164, and Pedometer Omron HJ-720IT. PA monitors were worn simultaneously for 1 week. Participants reported preferences and problems (irritating, cumbersome, movement of the activity monitor, technical difficulties, and impaired functioning) encountered with each activity monitor.

**Results:** Approximately 70% of the participants (*n* = 66) were middle-aged Caucasian women with a diagnosis of BD I and overweight. Sixty-six adults with BD wore all 3 monitors simultaneously. Twelve (18%) participants had no PA monitoring preference, 28 (42%) preferred the armband, 17 (26%) preferred the pedometer and 9 (14%) preferred the Actigraph. Activity monitoring preferences did not statistically differ by age, gender, race, BMI, diagnosis, or depressive and mania symptoms (*p* > 0.25). Two-thirds of the participants (64%) had at least one problem with at least one activity monitor. As far as problem categories, more than a quarter of participants reported irritation with the Armband (26%, *n* = 17) and movement of the pedometer (32%, *n* = 21). No statistically significant association was observed between activity monitoring preferences and problems (*p* = 0.72).

**Discussion:** Adults with BD were willing to wear activity monitors even though problems were reported. Preference of physical activity monitors, in descending order, was the armband, pedometer, and Actigraph. One fifth of the adults with BD reported no preferences in activity monitors. The activity monitors selected for investigation included the “gold standard” in activity monitoring (Actigraph) worn at the waist as well as a research grade pedometer that is considerably more affordable, provides activity feedback in real-time, and may be a more feasible option for large scale studies.

Adults with bipolar disorder (BD) are significantly less active and more sedentary compared to the general population and other users of mental health services (1). Physical inactivity is known to contribute to obesity and metabolic syndrome resulting in premature mortality (2) in spite of healthier eating habits (3). The Second Edition of the “Practice Guideline for the Treatment of Patients with Bipolar Disorder” by the American Psychiatric Association (4) did not contain recommendations about the increase of physical activity in patients with BD. The National Collaborating Centre for Mental Health (5) concluded that there was insufficient evidence about the effectiveness of physical activity in patients with BD. Four years later, Sylvia and associates (6) suggested, that exercise has beneficial effects for patients with BD. In 2013, Sylvia and associates (7) showed in a small pilot study positive effects of lifestyle intervention including physical activity on the mood of patients with BD that are obese. There is a growing demand on studies showing the relationship between BD on physical activity and vice versa. Physical activity studies in patients with BD are still very scarce. It is therefore unknown which types of activity monitors are preferred in patients with BD in order to maximize adherence/compliance to the study/intervention and minimize expenses. Physical activity can be assessed by self-report, by direct observation or by objective measurement. The latter have become very popular, given their easy administration and accuracy. Objective measurements include accelerometers (e.g., ActiGraph), pedometers, heart rate monitors and armbands (e.g., Body Media), which all may come in different forms and shapes (8). To date there are no studies available that report activity monitor preferences by patients with BD.

## Purpose

The Physical Activity and Function in Adults with Bipolar Disorder (PARC2) (1) study was designed to provide a profile of objectively measured physical activity among adults diagnosed with BD. At the time of study design and data collection, published studies of adults with BD had relied on only self-reported rather than objective measures of physical activity. The primary purpose of this secondary analyses was to determine activity monitor (waist actiGraphy, research grade pedometer or BodyMedia armband) preferences among patients with BD, and to identify problems associated with each activity monitor from the perspective of the patient with BD. Additional analyses included exploring the associations between activity monitor preferences and age, race, gender, level of depression, level of mania, and type of BD.

## Methods

Secondary data analysis was performed from the Physical Activity and Function in Adults with Bipolar Disorder (PARC2 study) (1) a cross sectional study of patients diagnosed with BD and treated for BD at the Western Psychiatric Institute and Clinic (WPIC) at the University of Pittsburgh, PA. Participant recruitment took place between January 2009 to November 2011 of individuals who were receiving treatment for BD at Western Psychiatric Institute and clinic (WPIC) at the University of Pittsburgh and were age ≥18 years. Patients were eligible for the study regardless of their clinical status (euthymic, depressed, hypomanic, or manic) or bipolar subtype (BD I, BD II, BD Not Otherwise Specified (NOS), BD NOS/Schizoaffective (SA) disorder). All research procedures were approved by the Institutional Review Board at the University of Pittsburgh. All researchers received training in and adhered to both institutional safeguards and Good Clinical Practice guidelines for the protection of human subjects. PARC2 participants signed informed consent documents prior to engaging in research procedures. Participants were compensated $10 from January 2009 to July 2011 and $30 from August 2011 to November 2011.

Briefly, PARC2 participation involved two clinic visits scheduled 1 week apart. At study entry, participants received at least one activity monitor (ActiGraph AM-7164 monitoring device, BodyMedia SW Pro Armband, and/or the Pedometer Omron HJ-720IT) and completed self-assessments. One week later, participants returned the activity monitors and completed additional self-assessments at the second clinic visit. At the second clinic visit, the participant's mood symptoms for the prior 7 days were assessed by independent evaluators using the 17-item Hamilton Depression (HRSD17) scale (9) and an expanded 25-item Hamilton Depression (HRSD25) scale that included reverse neurovegetative symptoms (10). The HRSD17 and HRSD25 scores range from 0–52 to 0–72, respectively, with higher scores indicating a greater burden of depressive symptoms. HRSD17 scores were categorized as not depressed ( ≤ 7); mild depressive symptoms (8–13); moderate depressive symptoms (14–19); severe depressive symptoms (≥20). Mania/hypomania was assessed with the Young Mania Rating Scale (YMRS) (11) with scores ranging from 0–60 and higher scores indicating higher levels of mania/hypomania. YMRS symptoms were categorized as not experiencing mania ( ≤ 6); mild mania (7–14); moderate mania (15–19); and severe mania (≥20). Psychiatric diagnoses (BD I, BD II, BD NOS, BD NOS/SA) were obtained from participants' research records in accordance with DSM IV criteria using the Structured Clinical Interview for the Diagnostic and Statistical Manual of Mental Disorders (SCID) Fourth edition (12) or Mini International Neuropsychiatric Interview (MINI) (13).

At the second clinic visit, participants answered several questions regarding which activity monitor they preferred and whether or not they experienced any problems with each activity monitor. In an open-ended comment field, the researcher recorded participant's stated problems with each activity monitor. The open-ended comments were reviewed, and common themes were categorized into the following 5 categories: irritating, cumbersome, moving, technical difficulties, and impaired functioning. Categories were assigned from either specific wording used in the comment sections or subjective wording describing an events that happened when wearing each activity monitor.

### Activity Monitors

Participants wore to 1 to 3 activity monitors. All participants wore the research-grade pedometer. Only study participants not enrolled in the Medrisk Study (14) were eligible to wear 2 additional activity monitors (Actigraph and BodyMedia Armband), if available, as described below.

#### Pedometer (Hip)

All study participants received a research-grade pedometer (model: Omron HJ-720IT; Omron, Bannockburn, IL) to objectively measure physical activity for 1 week. The pedometer was clipped on the elasticized belt provided for the actigraph and worn over the non-dominant hip. Participants were instructed to wear the pedometer during their waking hours for 7 consecutive days and to remove the pedometer for any water activities e.g., showers, and swimming. Tape masked the step counts displayed on the pedometer during the monitoring period.

#### Actigraph (Waist)

PARC2 replicated the NHANES 2003–2004 physical activity monitoring protocol for actigraphs (Department of Health and Human Services Center for Disease Control and Prevention, 2006). Briefly, participants were instructed to wear the ActiGraph AM-7164 monitoring device (ActiGraph, Ft. Walton Beach, FL) on an elasticized belt over the non-dominant hip for seven consecutive days. Actigraphs were not worn while sleeping or during water activities such as showering or swimming.

#### Armband

The study participants were instructed to wear the BodyMedia FIT Activity Monitor 24/7 on their left upper arm, directly on the skin, that was held in place with an elastic belt. The BodyMedia armband was only removed for water activities such as bathing/showering or swimming.

### Data Analysis

The analytical dataset for activity monitor preferences was restricted to study participants that wore 3 pedometers simultaneously (*n* = 66) and completed the activity monitor preference survey. Participants were classified into one of four groups based on activity monitor preference: Armband, Pedometer, Actigraph and None. Chi-squared and Fisher Exact tests were used to compare the activity monitor preferences with demographic variables and reported activity monitoring problems. Problems with the activity monitors were reported for the larger sample (*n* = 81) that completed an activity monitor preference survey and wore at least two monitors. Statistical analyses were performed using SAS (version 9.4, SAS Institute, Triangle Park, NC).

## Results

Eighty-one of the 101 PARC2 participants completed the activity monitor preference survey, a later addition to the protocol. Of the 81 participants, 76 wore an armband, 80 wore a pedometer, and 77 wore an actigraph. Sixty-six participants wore all 3 monitors simultaneously and constituted the analytical dataset (Table 1).

**Table 1:** Association between activity monitor preferences and demographics among adults with bipolar disorder enrolled in the PARC2 study (*n* = 66).

**Variable**		**Activity Monitor Preferences for Adults with Bipolar Disorder**				***p*-value statistical test**
	**Total Sample (*n* = 66)**	**Armband (*n* = 28)**	**Pedometer (*n* = 17)**	**Actigraph (*n* = 9)**	**No Preference (*n* = 12)**	
**Age (years)**						
Mean ± SD	44.3 ± 120	42.3 ± 13.9	43.8 ± 10.7	50.4 ± 8.8	45.0 ± 10.5	0.25^a^
Range	18.7–63.1	18.7–63.1	21.6–58.4	39.6–62.3	28.6–57.1	
**Gender** ***n*** **(%)**						
Female	45 (68.2)	19 (67.9)	13 (76.5)	5 (55.6)	8 (66.7)	0.73^b^
Male	21 (31.8)	9 (32.1)	4 (23.5)	4 (44.4)	4 (33.3)	
**Race** ***n*** **(%)**						
Asian	1 (1.9)	0 (0)	1 (6.7)	0 (0)	0 (0)	0.33^b^
Black	13 (25.0)	4 (18.2)	3 (20.0)	1 (20.0)	5 (50.0)	
White	38 (73.0)	18 (81.8)	11 (73.3)	4 (80.0)	5 (50.0)	
**BMI**						
Mean ± SD	29.5 ± 7.4	28.2 ± 7.8	29.7 ± 7.0^c^	34.5 ± 7.68	28.6 ± 5.6	0.32^a^
Range	18.9–46.5	19.3–46.0	18.9–46.5	24.7–43.8	22.7–44.0	
**Diagnosis** ***n*** **(%)**						
Bipolar I	44 (67.7)	20 (74.1)	12 (70.6)	4 (44.4)	8 (66.7)	0.26^b^
Bipolar II	17 (26.2)	7 (25.9)	4 (23.5)	4 (44.4)	2 (16.7)	
Bipolar NOS/SA	4 (6.2)	0 (0)	1 (5.9)	1 (11.1)	2 (16.7)	
**HRSD17**						
Mean ± SD	8.5 ± 6.4	9.1 ± 7.5	6.5 ± 4.4	10.8 ± 3.5	8.4 ± 7.4	1.00^a^
Range	0–31	0–31	0–12	6–17	0–24	
**HRSD25**						
Mean ± SD	11.5 ± 8.5	12.3 ± 10.0	8.8 ± 6.1	13.8 ± 3.6	11.5 ± 9.8	0.99^a^
Range	0–42	0–42	0–18	8–18	0–34	
**Young Mania Rating Scale**						
Mean ± SD	3.4 ± 4.1	3.3 ± 4.5	3.2 ± 4.7	5.2 ± 3.2	2.3 ± 2.5	0.86^a^
Range	0–20	0–20	0–12	0–10	0–7	

a *ANOVA performed to compare demographic differences between 4 preference groups (armband, pedometer, actigraph or no preference)*.

b *Fisher's Exact test to compare demographic differences between 4 preference groups (armband, pedometer, actigraph or no preference)*.

c *N = 15*.

*HRDS-17: Hamilton rating depression scale 17-item*.

*HRDS-25: Hamilton rating depression scale 25-item*.

*Bipolar NOS/SA, bipolar disorder not otherwise specified/schizoaffective disorder*.

**Figure 1:**
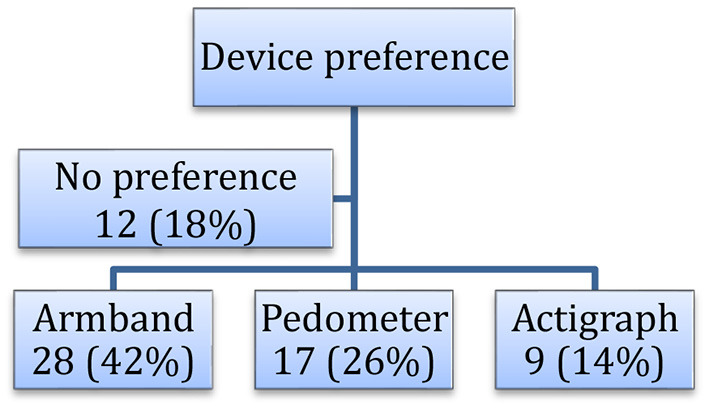
Preferences of physical activity monitoring devices in adults with bipolar disorder (*n* = 66) in the PARC2 study.

Approximately 70% of the participants were middle-aged Caucasian women with a diagnosis of BD I and overweight (Table 1). Overall, the armband (42%) and actigraph (14%) were the most and least preferred activity monitors, respectively (Figure 1). Twelve participants (18%) reported no preference for any activity monitor. Activity monitoring preferences did not statistically differ by age, gender, race, BMI, diagnosis, or depressive and mania symptoms (*p* > 0.25 No statistically significant association was observed between activity monitoring preferences and problems (*p* = 0.72).

**Figure 2:**
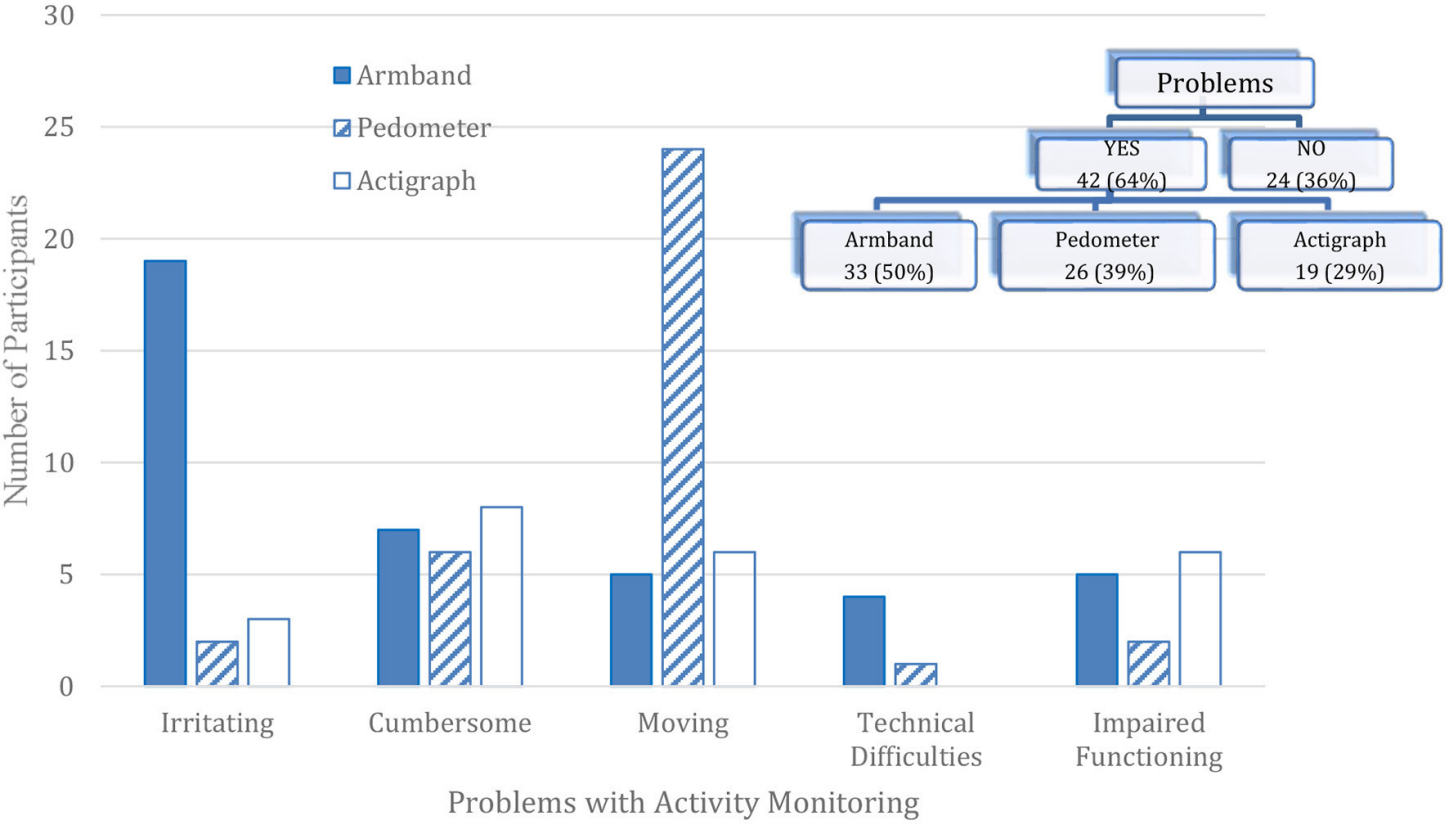
Activity monitor problems reported among adults with bipolar disorder enrolled in the PARC2 study (*n* = 66).

Two-thirds of the participants (64%) had at least one problem with at least one activity monitor (Figure 2) ranging from 29% for the Actigraph to 50% for the Armband. As far as problem categories, more than a quarter of participants reported irritation with the Armband (29%, *n* = 19) and movement of the pedometer while wearing it (36%, *n* = 24). Pedometer movement or falling off may have been due to the pedometer being clipped on the elasticized belt for the actigraph rather than clipped on a waistband or pocket.

## Discussion

The PARC2 study provided the first profile of objectively measured physical activity and sedentary behavior in adult outpatients with BD. This study supplements those findings by investigating activity monitoring preferences and problems among adults with BD. Almost half of the participants preferred the Armband even though many indicated that it was irritating. Next, a quarter of the participants preferred the pedometer. The primary problem with the pedometer was movement or the pedometer falling off the elasticized nylon belt. Although speculative, the movement problem may not have occurred if the pedometer was worn alone and clipped to a waistband, pocket, or traditional leather belt. Instead, the pedometer was clipped to the Actigraph's elastic band which may have resulted in noticeable movement when worn by the participant. Interesting, the Actigraph worn at the waist was the least preferred activity monitor even though the Actigraph had the fewest number of reported problems.

It should be emphasized that none of the activity monitors provided activity feedback to the participant during the monitoring period. It is possible that preferences for the pedometer and Armband may have been even higher if they provided the daily and ongoing feedback as intended. Anecdotally, some participants actively concealed the activity monitors under their clothing, and this concern may have contributed to their activity monitor preference selection. In addition, this study was conducted prior to wristbands such as Fitbits. Speculatively, the primary problems (irritation and movement) observed in the armband and the pedometer would be unlikely problems with wrist bands. However, activity monitors worn as wristbands may have other problems and should be further investigated.

## Conclusion

Overall, adults with BD were willing to wear activity monitors even though problems were reported. The activity monitors selected for investigation included the “gold standard” in activity monitoring (Actigraph) worn at the waist as well as a research grade pedometer that is considerably more affordable, provides activity feedback in real-time, and may be a more feasible option for large scale studies. Future analyses should investigate the quantitative differences in the objective measures of physical activity obtained from the activity monitors, and the association between objective and self-reported measures of physical activity among adults with BD.

## Data Availability Statement

The raw data supporting the conclusions of this article will be made available by the authors, without undue reservation.

## Ethics Statement

The studies involving human participants were reviewed and approved by University of Pittsburgh Institutional Review Board. The patients/participants provided their written informed consent to participate in this study.

## Author Contributions

CJ and AF designed and conducted the study. CJ analyzed the data. AD, RR, and MS wrote the first draft of the manuscript with Dr. Janney's mentorship. All authors contributed to the article and approved the submitted version.

## Conflict of Interest

CJ received research support to compensate study participants from Actigraph, Inc. AF is /has been a consultant and/or a speaker and/or has received research grants from Angelini, Apsen, Boheringer Ingelheim, Daiichi Sankyo, Doc Generici, Glaxo Smith Kline, Lundbeck, Janssen, Mylan, Neuraxpharm, Otsuka, Pfizer, Recordati, Sanofi Aventis, Sunovion, Vifor. The remaining authors declare that the research was conducted in the absence of any commercial or financial relationships that could be construed as a potential conflict of interest.
